# Clear Cell Renal Carcinoma in an Ulcerative Colitis Patient Under Short-Term Immunosuppressive Therapy: A Case Report

**DOI:** 10.3390/clinpract15040075

**Published:** 2025-04-03

**Authors:** Raffaele Pellegrino, Giuseppe Imperio, Michele Izzo, Ilaria De Costanzo, Fabio Landa, Paola Ciamarra, Marco Niosi, Antonietta Gerarda Gravina, Alessandro Federico

**Affiliations:** Hepatogastroenterology Division, Department of Precision Medicine, University of Campania Luigi Vanvitelli, Via L. de Crecchio, 80138 Naples, Italy

**Keywords:** ulcerative colitis, renal cancer, immunosuppression, inflammatory bowel disease, filgotinib, infliximab

## Abstract

**Background/Objectives:** Renal cell cancer is a rare occurrence in patients with ulcerative colitis (UC), with no clearly demonstrated association between UC and an increased risk of renal malignancies. In this article, a case report concerning this relationship is presented. **Methods:** Our research group presented a case of clear cell renal carcinoma in a 56-year-old male with UC who had previously undergone ileorectal anastomosis and subtotal colectomy. **Results:** The patient developed a complex renal cyst that progressed to malignancy within one year while on immunosuppressive therapy with infliximab and then filgotinib. Previous ultrasound examinations of the kidney highlighted only simple cysts in the contralateral kidney in previous years. The neoplasm was promptly examined using contrast-enhanced ultrasound, confirming the diagnosis of a Bosniak IV cyst, which was corroborated by a subsequent computed tomography study. **Conclusions:** The patient underwent a nephrectomy and is currently scheduled for therapy with vedolizumab. Given the increasing use of biologics and small molecules in UC management, periodic ultrasound screening may be a valuable tool for the long-term monitoring of these patients.

## 1. Introduction

Ulcerative colitis (UC) belongs to the group of inflammatory bowel diseases (IBDs) and induces a chronic, self-perpetuating inflammatory condition affecting the colorectal tract, starting from the rectum, and this condition exposes patients to both short- and long-term complications, including colectomy and colorectal cancer [1]. In addition to colorectal cancer, which has a prevalence of nearly 4% among UC patients and for which they face a significantly higher risk compared to the general population, other forms of neoplastic associations have also been described, including renal cancer [2].

In this regard, Feng et al. [3], in a pooled analysis of population-based studies, demonstrated an increased risk of renal cancer in patients with IBD (pooled standardised incidence ratio of 1.53), particularly in those with Crohn’s disease (pooled standardised incidence ratio of 1.95). However, no increased risk was observed in UC patients compared to the general population [3]. This risk profile was further confirmed by a subsequent reanalysis of meta-analyses conducted by Piovani et al. [4], which ruled out any defined associations between renal cancer and UC. Conversely, data from Mendelian randomization genetic studies have failed to demonstrate a potential causal association between IBD and renal cancer [5].

Advanced immunosuppressive therapies for IBD, including biologics and small molecules, have demonstrated—albeit with a low level of evidence and considerable heterogeneity among the limited studies conducted—a variable association with different malignancies [6]. Cases of renal cell carcinoma in patients with UC are rare [7,8,9] and tend to occur at a younger age, with better survival outcomes irrespective of immunosuppressive therapy use [10].

This report highlights the rare onset, in the context of UC, of renal cancer arising from a simple renal cyst background, developing from a complex cyst in the short-term following immunosuppressive treatment with a biologic agent.

## 2. Case Presentation

Our research group reports the case of a UC 56-year-old male patient (diagnosis in 2011) who underwent colectomy with ileorectal anastomosis. In December 2022, a complete abdominal ultrasound revealed only bilateral simple renal cysts (maximum diameter of 18 mm in the left kidney) and fatty liver.

Additionally, the patient’s medical history included osteoporosis complicated by vertebral collapse, as well as grade B oesophagitis according to the Los Angeles classification, which was managed with acid-suppressive therapy.

Following surgery, the patient was placed on oral maintenance therapy with mesalamine at a dosage of 2.4 g/day, combined with intermittent cycles of topical mesalamine suppositories. However, adherence to topical therapy was inconsistent due to suboptimal patient compliance. The subsequent development of steroid dependency necessitated consideration of the introduction of biological therapy.

In March 2023, he began treatment with infliximab (at a dose of 5 mg/kg using the standard regimen for UC) due to severe endoscopic activity (Mayo endoscopic score 3) and lack of compliance with further surgery. Before the introduction of infliximab, the patient underwent infectious disease screening, which was negative (i.e., TORCH complex, QuantiFERON for tuberculosis), as well as a complete abdominal ultrasound. The ultrasound revealed a bright liver with a homogeneous hepatic structure and mild splenomegaly and confirmed the presence of simple bilateral renal cysts with a stable overtime maximum diameter of 18 mm in the left kidney.

Seven months later, the patient switched to filgotinib due to a failure to maintain clinical remission (i.e., secondary loss of response).

In April 2024, the patient underwent an endoscopic reassessment for persistent clinical disease activity, which confirmed rectal endoscopic activity (as per Mayo endoscopic score 3). Additional medical tests were conducted during the general reassessment of the patient, summarised in Table A1. The patient also underwent an essential cardiological evaluation with an electrocardiogram, demonstrating a sinus rhythm at a heart rate of 78 bpm (PR interval within normal limits, QRS electrical axis normally oriented, QRS within normal limits, no significant abnormalities in ventricular repolarisation). In addition, the patient underwent a microbiological rectal and pharyngeal-tonsillar swab, both of which resulted negative.

**Figure 1:**
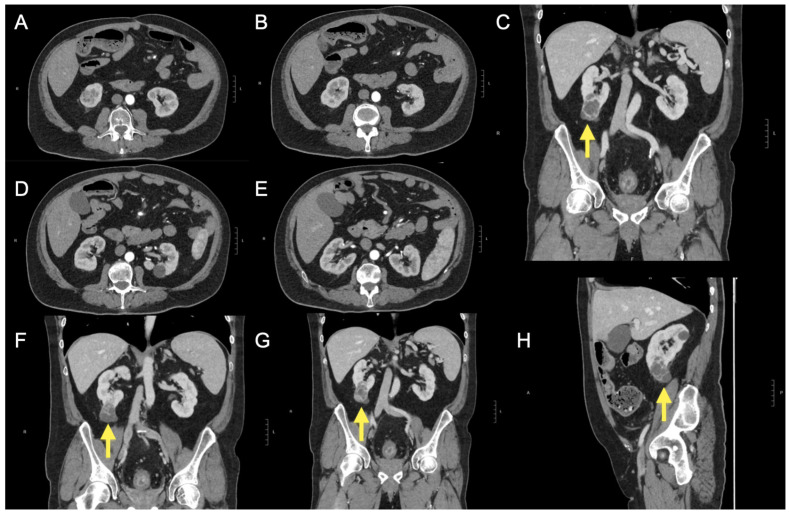
After the ultrasound findings, a computed tomography (CT) scan was performed (**A**–**H**) with and without intravenous contrast administration (iopromide 370 mg/mL). At the lower pole of the right kidney, the scan revealed a complex cystic lesion characterised by multiple thick septa and several small nodular projections (**C**,**F**,**G**,**H**), which demonstrated contrast enhancement. The lesion measured approximately 38 × 32 × 55 mm (anteroposterior, transverse, and craniocaudal diameters). These findings are consistent with a Bosniak category IV cyst. The lesion did not infiltrate vascular structures or the renal sinus. The left kidney was unremarkable. The excretory pathways were not dilated. Additional simple cortical cysts were noted bilaterally in the kidneys. No significant abnormalities were detected in other abdominal structures, except for two small hypodense lesions located subcapsularly in the liver, at the fifth and anterior fourth segments, likely cystic. The CT scan also demonstrated diffuse and concentric thickening of the rectal walls, indicative of chronic inflammatory changes and a few sub-centimetric mesorectal lymph nodes. The yellow arrow further helps to identify the renal lesion described.

During the same evaluation, an abdominal ultrasound revealed a 45 mm cystic lesion with septa at the lower pole of the right kidney. Upon power Doppler and microvascular blood imaging ultrasound evaluation, the septa appeared vascularised. CEUS evaluation demonstrated hyperenhancement, raising suspicion for cystic renal neoplasia. Further investigation with contrast-enhanced CT of the abdomen (Figure 1) confirmed the presence of a Bosniak IV complex renal cyst.

The multidisciplinary evaluation recommended a laparoscopic right nephrectomy, which the patient underwent in June 2024 with an uneventful postoperative course. A histopathological examination revealed a clear cell renal carcinoma with a maximum lesion diameter of 15 mm, graded as G2 according to the WHO/ISUP system. There was no infiltration of the renal sinus fat or renal capsule, corresponding to a pT1a stage according to the 8th edition of the AJCC/TNM classification. Renal function indices were consistently within normal limits before the diagnosis of the complex cyst, and in the reports from our institution since 2022, there has been no flag indicating renal function impairment or alterations in the related haematochemical parameters.

We will evaluate with the oncologist, surgeon, and patient the continuation of therapy for UC, considering surgery or, if declined, continuing with biologics with a top safety profile (e.g., vedolizumab).

## 3. Discussion

### 3.1. What are the Possible Considerations in This Case Report?

This clinical case reported the rare onset of a renal neoplasm based on a complex renal cyst that developed over approximately one year in a patient with a previously documented absence of related lesions. The patient had undergone prior treatment with infliximab and was receiving active treatment with filgotinib for ulcerative proctitis with severe endoscopic activity, steroid-dependent, following ileorectal anastomosis and subtotal colectomy. In other terms, this case highlights the potential oncogenic risk of chronic immunosuppression in immune-mediated diseases. It also underscores the value of a non-invasive investigation such as the US, which identified this complex cyst (harbouring clear cell renal carcinoma) that evolved significantly in size and characteristics over a year (on a background of bilateral simple renal cysts).

As previously mentioned, the reported and well-documented cases of renal cancer in patients undergoing immunosuppressive treatment for UC are not numerous. In a series from the 1990s, Satsangi et al. [8] described three cases of renal cancer in patients with an oncological diagnosis occurring between seven and fourteen years after the diagnosis of UC, all of whom had been treated with azathioprine.

Casellas et al. [7] instead described two cases of renal cancer, one in a young 37-year-old patient treated only with oral sulfasalazine and topical steroids and another in a 53-year-old patient treated with steroids and oral sulfasalazine but with a history of chronic renal failure as an underlying condition.

Moreover, cases of acute severe UC have also been described, induced by the concurrent presence of renal cancer, triggering a condition similar to toxic megacolon due to mass effect, as in the report by Kukulska et al. [11]. However, whether the patient described had previously undergone immunosuppressive therapy is not specified.

In addition, beyond UC, Zarur et al. [12] described two cases of renal cancer arising during immunosuppressive therapy for psoriasis. One case involved a 66-year-old patient treated with methotrexate and etanercept who was diagnosed with papillary renal cancer approximately seven months after starting treatment. The other case involved a 41-year-old man treated with methotrexate and infliximab, who was diagnosed with renal cancer about four months after initiating therapy.

### 3.2. The Risk of Renal Cancer During Immunosuppressive Therapy in IBD

As previously stated, the current evidence does not explicitly demonstrate an increased baseline risk of renal cancer in patients with UC [3,4]. It is therefore plausible that factors related to chronic therapeutic immunomodulation may modify this risk.

Nevertheless, a large meta-analysis of randomised controlled trials has already demonstrated that the use of anti-TNF agents in patients with IBD showed a prevalence of neoplasia of 0.39% among the more than four thousand patients analysed, with a relative risk of cancer compared to placebo of 0.77 (95% CI 0.37–1.59), which was not statistically significant [13]. This finding indicates no association with an increased risk of malignancy, albeit within the limitation of a follow-up period generally confined to approximately one year of treatment [13]. On the other hand, for the filgotinib class (i.e., JAK inhibitors), Olivera et al. [14] conducted a meta-analysis of trials, confirming the absence of an increased cancer risk for this treatment class. This finding was further confirmed in a subsequent meta-analysis [15]. Strong real-world practice data are also available for infliximab, which has been used for decades compared to JAK inhibitors. These data, distinct from those of randomised controlled trials, have confirmed the absence of a clear association between these biological agents and cancer risk [16].

In addition, this report highlights how the diagnosis of neoplasia can occur even after short-term use of immunosuppressants, whereas in other cases, tumours have been reported only after several years of use [17,18]. However, albeit rare, cases have also been documented after just a few months [19].

Some evidence can also be extrapolated from settings not explicitly related to IBD. Ilham et al. [20] also conducted a large retrospective cohort study on over thirteen thousand patients, including recipients of solid organ transplants or hematopoietic stem cell transplants, patients diagnosed with primary or secondary immunodeficiency disorders, and those receiving anti-TNF agents (the latter constituting 24% of the sample). In this large cohort, they identified a cumulative cancer incidence of 11.5% (specifically 8.8% in the group treated with anti-TNF agents). Interestingly, patients with renal cysts (HR 2.54) had an increased risk of developing cancer in general compared to those without. Moreover, having a renal cyst can increase the overall risk of renal cell carcinoma [21,22]. Nonetheless, when examining cohort studies in populations of solid organ transplant recipients, such as the US Scientific Registry of Transplant Recipients (1987–2008) involving over 170,000 patients, a cancer incidence of 1375 per 100,000 person-years was identified, with a specific incidence for kidney cancer of 97 per 100,000 person-years [23].

### 3.3. Case Report Limitations

By its very nature, this report is limited by the description of a single case, which is a consequence of the condition’s rarity, making it challenging to organise larger-scale studies with a greater sample size. Furthermore, no specific molecular assessments were conducted on the tumour regarding the signalling pathways related to filgotinib and infliximab. However, this is understandable given the observational nature of the report and the fact that the procedures performed were those pertaining to standard clinical practice. Clearly, this report cannot demonstrate a direct causal link between the administered drugs and the onset of cancer but merely describes an epidemiological event that occurred.

## 4. Conclusions

In conclusion, all these cumulative data demonstrate the rarity of a malignant renal neoplasm diagnosis in the context of IBD treated with biological agents or small molecules. However, although rare and isolated, cases can still occur; this underscores the importance of implementing long-term monitoring for these patients using basic, non-invasive, cost-effective, and repeatable tools, such as ultrasound examinations. These provide a broader abdominal overview, not solely focused on the gastrointestinal tract, as with endoscopy and intestinal radiology techniques. Especially in the current context, where intestinal ultrasound also provides the opportunity to assess the small intestine and colon effectively, and now, with transperineal ultrasound, even the rectum [24,25,26,27].

## Data Availability

The original contributions presented in this study are included in the article. Further inquiries can be directed to the corresponding author.

## Appendix A

**Table A1:** Haematochemical evaluations of the patient related to the reassessment in April 2024.

Parameter	Value	Normal Values	Unit of Measurement
WBC	6.28	4–11	10^3^/µL
RBC	4.61	4.5–6	10^6^/µL
RET	0.17	0.025–0.075	10^6^/µL
HGB	7.9	13–17.5	g/dL
HCT	29.8	42–51	%
MCV	64.6	82–96	fL
MCH	17.1	27–31	pg
RDW-SD	43.7	36–43	fL
PLT	384	150–450	10^3^/µL
PT	10.6	9–13	s
INR	0.92	0.8–1.2	N.A.
aPTT	27.8	24–38	s
Fibrinogen	484	200–450	mg/dL
Blood glucose	92	70–100	mg/dL
Urea	37	10–50	mg/dL
Creatinine	0.84	0.67–1.17	mg/dL
Uric acid	4.6	3.4–7	mg/dL
Na^+^	141	135–146	mEq/L
K^+^	4.2	3.5–5.3	mEq/L
Cl^-^	108	98–111	mEq/L
Mg^2+^	1.9	1.6–2.6	mg/dL
Ca^2+^	9	8.6–10.2	mg/dL
ALT	15	5–41	U/L
AST	16	5–40	U/L
Amylase	94	28–100	U/L
Lipase	34	13–78	U/L
Alkaline phosphatase	58	40–129	U/L
CPK	51	60–190	U/L
LDH	153	120–240	U/L
CRP	1.9	0–1	mg/dL
Cholinesterase	8241	5320–12,920	U/L
Total bilirubin	0.36	<1.2	mg/dL
Conjugated bilirubin	0.14	<0.3	mg/dL
Unconjugated bilirubin	0.22	<0.75	mg/dL
Total proteins	6.9	6.6–8.7	g/dL
Albumin	3.9	3.5–5.5	g/dL
Serum iron	14	59–158	µg/dL
Ferritin	2	30–400	ng/dL
Transferrin	315	191–337	mg/dL
ESR	34	2–15	mm/h
IgA	313	70–400	mg/dL
IgG	1210	700–1600	mg/dL
IgM	138	40–230	mg/dL
Anti-endomysial IgA	Absent	Absent	-
IgA anti-Tg	3.2	<20	U/mL
IgG anti-Tg	3.2	<9	U/mL
HBsAg	0.03	<0.05	IU/mL
HBsAb	<3	>9	mUI/mL
HBcAb	Absent	Absent	-
HBeAb	Absent	Absent	-
HCVAb	Absent	Absent	-
Vitamin B_12_	275	187–883	pg/mL
HDL	30	>35	mg/dL
LDL	94	10–129	mg/dL
Total cholesterol	164	60–200	mg/dL
QuantiFERON-TB Gold plus test for Mycobacterium tuberculosis: IFN-γ after stimulation with TB-specific antigens TB1 (CD4)	0	<0.35	UI/mL
QuantiFERON-TB Gold plus test for Mycobacterium tuberculosis: IFN-γ after stimulation with TB-specific antigens TB2 (CD4/CD8)	0	<0.35	UI/mL
QuantiFERON-TB Gold plus test for Mycobacterium tuberculosis: IFN-γ after stimulation with mitogen	9.9	≥0.5	UI/mL

**Note**: WBC: white blood cells; RBC: red blood cells; RET: reticulocytes; HGB: haemoglobin; HCT: haematocrit; MCV: mean corpuscular volume; MCH: mean corpuscular haemoglobin; RDW-SD: red cell distribution width—standard deviation; PLT: platelets; PT: prothrombin time; INR: international normalised ratio; aPTT: activated partial thromboplastin time; ALT: alanine aminotransferase; AST: aspartate aminotransferase; CPK: creatine phosphokinase; LDH: lactate dehydrogenase; CRP: C-reactive protein; ESR: erythrocyte sedimentation Rate; IgA: immunoglobulin A; IgG: immunoglobulin G; IgM: immunoglobulin M; Tg: transglutaminase; HBsAg: hepatitis B surface antigen; HBsAb: hepatitis B surface antibody; HBcAb: hepatitis B core antibody; HBeAb: hepatitis B envelope antibody; HCVAb: hepatitis C virus antibodies; HDL: high-density lipoprotein; LDL: low-density lipoprotein.
